# Measuring contraceptive self-efficacy in women: the Turkish validity and reliability study

**DOI:** 10.1017/S146342362500026X

**Published:** 2025-05-06

**Authors:** Aliye Dogan Gangal, Ayten Senturk Erenel

**Affiliations:** 1 Faculty of Nursing, Department of Gynaecology and Obstetrics Nursing, Gazi University, Ankara, Türkiye; 2 Faculty of Health Science, Department of Nursing, Lokman Hekim University, Ankara, Türkiye

**Keywords:** Contraception, reliability, self-efficacy, validity

## Abstract

**Aim:**

This study aimed to evaluate the validity and reliability of the Turkish version of the Contraceptive Self-Efficacy in Women in Sub-Saharan Africa (CSESSA) scale.

**Background:**

Contraceptive self-efficacy is a crucial predictor of utilization of modern contraceptive methods. However, the existing tools for comprehensively assessing contraceptive self-efficacy are limited. Methods: The sample of this methodological study consisted of 510 female participants of reproductive age. The translation and cultural adaptation of the scale were performed. For validity, content validity and construct validity were tested. For reliability, test-retest reliability, Cronbach’s alpha coefficient, and item-total score correlations were evaluated. Findings: The goodness-of-fit indices showed an overall acceptable fit with the three-factor model. Cronbach’s alpha for the overall CSESSA scale was 0.867, and for the three subscales, it ranged from 0.77 to 0.84. The scale’s test-retest reliability was found to be r = 0.83 (p < 0.001), and the item-total correlations score ranged from 0.495 to 0.646. The Turkish version of the scale is a valid and reliable tool to measure the contraceptive self-efficacy of women of reproductive age. This scale can provide a comprehensive understanding of self-efficacy by assessing various dimensions of contraceptive self-efficacy.

## Background

Unintended pregnancies are a significant global public health issue (Bellizzi *et al.*, 2020; United Nations, 2019). It is known that the main reason behind unintended pregnancies is inconsistent or non-use of modern contraceptive methods (Finner & Zolna, 2016). Several factors influence the use of contraceptive methods. Some of these are economic factors associated with obtaining contraceptive methods, cultural and social factors that conflict with method use, and widespread negative beliefs. Additionally, lack of knowledge about contraception, including how to use contraceptive methods effectively and where to access them, negatively impacts method use (D’Souza *et al.*, 2022; Shah *et al.,* 2021; Yeh *et al.,* 2022).

Self-efficacy is important in the process of ending undesirable behaviours or promoting health-promoting behaviours (Bandura, 2005). For this reason, the use of contraceptive methods in a consistent manner requires behavioural changes and high self-efficacy (Lopez *et al.*, 2016; Uysal *et al.*, 2023). Research has indicated that women with high contraceptive self-efficacy demonstrate greater acceptance and compliance with contraceptive methods (Hamidi *et al.*, 2018; Shah *et al*., 2021). Evaluating contraceptive self-efficacy is crucial, as it is a significant predictor of modern contraceptive method use. There are few validated tools available for a comprehensive assessment of contraceptive self-efficacy (Brafford and Beck, 1991; Levinson *et al*., 1998; Richardson *et al.*, 2016; Bhan *et al.,* 2024). The validated tools that do exist often focus on condom use or include items adapted from other existing tools (Burke *et al.*, 2022; Hamidi *et al.*, 2018; Nelson *et al.*, 2017). Furthermore, these assessments often focus on ‘partner communication’, overlooking a more comprehensive perspective. Therefore, it is clear that using a comprehensive measurement tool is essential for assessing contraceptive self-efficacy accurately. Whiting-Collins *et al.* (2020) developed a measurement tool to evaluate the contraceptive self-efficacy of postpartum women and reported that it is valid and reliable in this population. It is considered that this short and easy-to-understand scale, which includes different dimensions of self-efficacy, can be used to measure the contraceptive self-efficacy of all women of reproductive age. To our knowledge, there is no measurement tool that evaluates the contraceptive self-efficacy of women with different dimensions. Therefore, examining the validity and reliability of this tool in Turkish is a guide for future studies. This study aimed to adapt the Contraceptive Self-Efficacy Among Women in Sub-Saharan Africa (CSESSA) scale into Turkish and to evaluate the validity and reliability.

## Methods

This methodological study tested the validity and reliability of the Turkish version of CSESSA Scale. This study adhered to the ‘International Test Commission Guidelines for Translating and Adapting Tests’ (International Test Commission 2017). There are different approaches to determining the sample size of validity and reliability studies (Anthoine *et al.*, 2014). To evaluate the factor structure of the scale, it is recommended to aim for a large number of participants (Boateng *et al.*, 2018; International Test Commission, 2017). In determining the sample size, Comrey and Lee (2013) provided the following guidance: 100 = poor, 200 = fair, 300 = good, 500 = very good, ≥ 1000 = excellent. Accordingly, this study aimed to reach at least 500 women. This study was conducted with 510 women. Participants had to meet the following inclusion criteria: Reproductive age, having a sexual partner, being literate.

**Figure 1.**
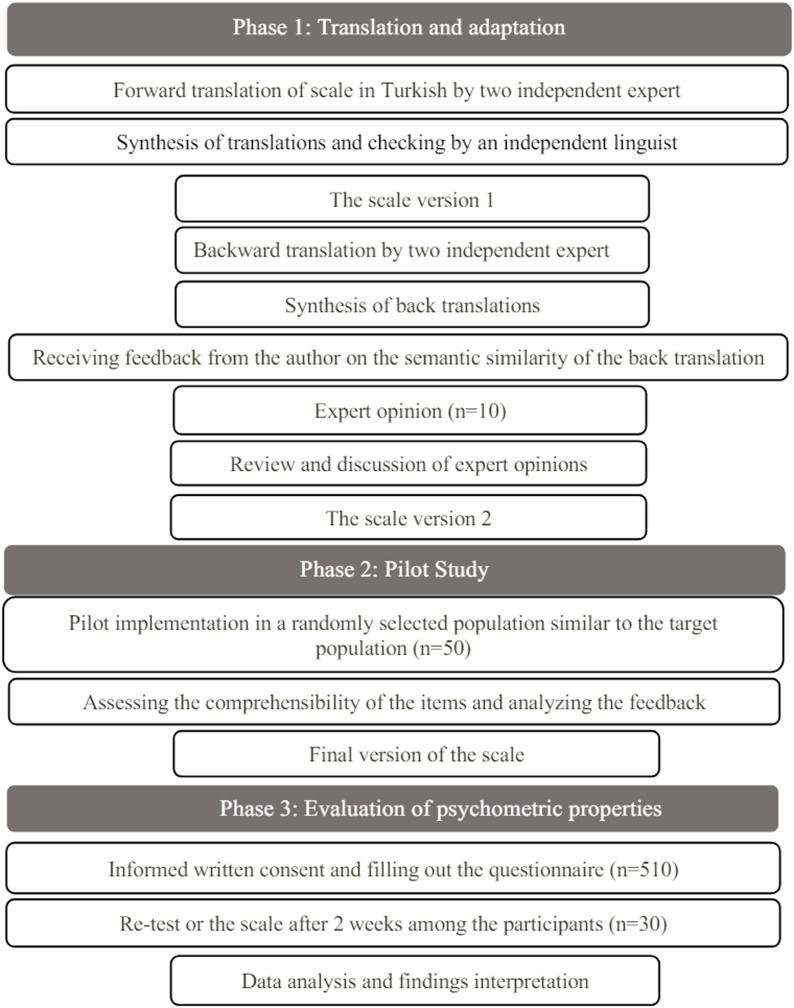
Stages of adaptation of the scale into Turkish.

This study was conducted in three stages. (1) Translation and adaptation of the scale, (2) pilot study, (3) evaluation of psychometric properties (Figure 1).

### Phase 1 Translation and adaptation

The first phase of the cross-cultural adaptation process is translation. Experts who are familiar with the terminology of the translated scale and who have experience collecting data on the subject take part in the translation phase (World Health Organization, 2017). Based on this recommendation, the scale was independently translated into Turkish by two experts with PhD degrees in gynaecology and obstetrics nursing. The two translations obtained were synthesized by the researchers, and an independent linguist checked the translations and analysed the sentence structures. In the subsequent stage, back-translation was performed by two independent translators with no knowledge of the original scale. After this stage, feedback was received from Whiting-Collins regarding the back-translation of the scale. It was decided that the translation of the scale items was appropriate and no items were removed. In the next stage, expert opinions regarding the scale items were obtained from 10 experts (gynaecology and obstetrics nursing, public health nursing, measurement, and evaluation experts). Modifications were made according to their feedback and the final form for the pilot study was obtained (Borsa *et al.*, 2012; Coster & Mancini, 2015).

### Phase 2 Pilot study

A pilot study was conducted to assess the validity of the scale. The scale was applied to 50 women who were similar to the target population (Borsa *et al.*, 2012; World Health Organization, 2017). The results of the pilot study indicated that all scale items were understandable, and no revisions were required.

### Phase 3 Evaluation of psychometric properties

Data were collected face-to-face from women admitted to primary health care centres in a province in Türkiye between May and December 2022. The data were collected by using a descriptive information form and the Turkish version of the CSESSA Scale. The baseline characteristics of 510 participants were analysed. Two weeks later, a retest was performed with 30 participants to evaluate test-retest reliability (Alpar, 2014). Confirmatory factor analysis (CFA) was performed to assess the construct validity of the scale, while test-retest reliability, item-total score correlations, and internal consistency analyses were carried out to evaluate its reliability.

## Data collection tools

### Descriptive information form

The researchers developed the form based on existing literature. It includes 15 items related to the participants’ demographic, obstetric, and contraceptive characteristics (Bellizzi *et al.*, 2020; Mutumba *et al.*, 2018; Upadhyay *et al.*, 2014).

### Contraceptive self-efficacy scale

The scale, developed in Kenya and Nigeria, measures the contraceptive self-efficacy of postpartum women. It consists of 11 items divided into three subscales: ‘husband/partner communication’, ‘provider communication’, and ‘choosing and managing a method’. Each item is scored on a visual analog scale from 0 (‘cannot do at all’) to 10 (‘highly certain can do’). Although the subscales can be scored independently, the total score is the sum of the subscale scores and ranges from 0 to 110. Higher scores indicate a higher contraceptive self-efficacy level. The original scale has Cronbach’s alpha of 0.90. The subscales have Cronbach’s alpha coefficients of 0.89, 0.89, and 0.88, respectively (Whiting-Collins *et al.*, 2020).

## Data analysis

The data were analysed using SPSS (v.22) and AMOS (v.21). For categorical data, we calculated the number and percentage. For quantitative data, we calculated the mean and standard deviation. Content and construct validity indices were examined to assess the scale’s validity. To assess content validity, the Davis technique was used, and 10 experts evaluated each item of the scale as ‘very appropriate’ (4 points), ‘appropriate but minor changes are needed’ (3 points), ‘the item needs to be transformed into an appropriate form’ (2 points) and ‘not appropriate’ (1 point). Item-Content Validity Index (I-CVI) and Scale Content Validity Index (S-CVI/Ave) were calculated (Polit and Beck, 2017). The I-CVI was calculated by dividing the sum of the items rated with three and four points by the total number of experts. S-CVI-Ave was calculated by dividing the sum of the I-CVI by the total number of items. The construct validity of the scale was tested by calculating Confirmatory Factor Analysis (CFA) goodness-of-fit indices. The following fit indices were evaluated to assess model fit: χ^2^/df (Chi-Square/Degree of Freedom), Comparative Fit Index (CFI), Goodness-of-Fit Index (GFI), Adjusted Goodness-of-Fit Index (AGFI), Tucker-Lewis Index (TLI), Normed Fit Index (NFI), and Root Mean Square Error of Approximation (RMSEA). The acceptable fit values for these indices are as follows: χ²/df <5, CFI ≥ 0.90, GFI ≥ 0.90, AGFI ≥ 0.85, TLI ≥ 0.90, NFI ≥ 0.90, and RMSEA ≤ 0.08. To assess reliability, we used Cronbach’s alpha coefficient to evaluate the internal consistency of the scale and its subscales (Polit and Beck, 2017). We also performed an item-total score analysis to examine the relationship between item scores and the total score. To determine time invariance, we carried out a test-retest and calculated the correlation coefficient (Streiner *et al.*, 2015). In addition, Hotelling’s T^2^ test was used to evaluate response bias and Tukey’s Test of Additivity was used to evaluate additivity. A p-value of less than 0.05 was considered to show a statistically significant result.

## Results

The study sample consisted of 510 participants aged between 20 and 53 years (mean=35.25 ± 6.58). All participants were married, and 37.1% held a university or master’s degree. Of all participants, 69.0% were unemployed, and 53.5% had a middle perceived economic level. Additionally, 91.4% of participants had at least one child, and 15.9% had a history of unintended pregnancy. While 70.8% reported not wanting to get pregnant, 32.7% did not use any modern contraceptive methods. Table 1 summarizes the baseline characteristics of the participants.

**Table 1.** Characteristics of participants (n = 510)

Characteristics		
Age (Mean ±SD) (Min-Max)	35.25 ± 6.58 (20–53)	
CSESSA scale (Mean ±SD)	9.03± 1.51	
Subscales of the Scale (Mean ±SD)		
Husband /partner communication	9.14 ±1.87	
Provider communication	8.92 ±1.91	
Choosing and managing a method	9.00 ±1.86	
	N	%
Education level		
Primary–Middle	156	30.6
High	165	32.4
Bachelor’s or Postgraduate	189	37.1
Employment		
Yes	158	31
No	352	69
Perceived economic level		
Low	153	30
Middle	273	53.5
High	84	16.5
Number of children		
0	49	9.6
1	91	17.8
2	230	45.1
3 and above	140	27.5
Unintended pregnancy history		
Yes	81	15.9
No	429	84.1
Pregnancy intention		
In the next 3 months	33	6.5
Within 1–2 years	45	8.8
Within 2–5 years	46	9.0
After more than 5 years	25	4.9
I don’t want to get pregnant	361	70.8
Contraceptive methods		
Modern Contraceptive Method*	343	67,3
Traditional method**	111	21.7
No method	56	11.0
Contraceptive method decision		
Partner decision	49	9.6
Own decision	148	29
Decision with partner	313	61.4

* Condom, pill, injections, spiral, subcutaneous implant, tubal ligation.

** Withdrawal, calendar.

The scale has total scores ranging from 0 to 110 points. The overall mean score, calculated by dividing the total score by the number of scale items, is 9.03 with a standard deviation (SD) of 1.51. The mean scores in sub-dimensions are also high: 9.14 (SD 1.87) for ‘husband/partner communication’, 8.92 (SD 1.91) for ‘provider communication’ and 9.00 (SD 1.86) for ‘choosing and managing a method’ (Table 1).

**Figure 2.**
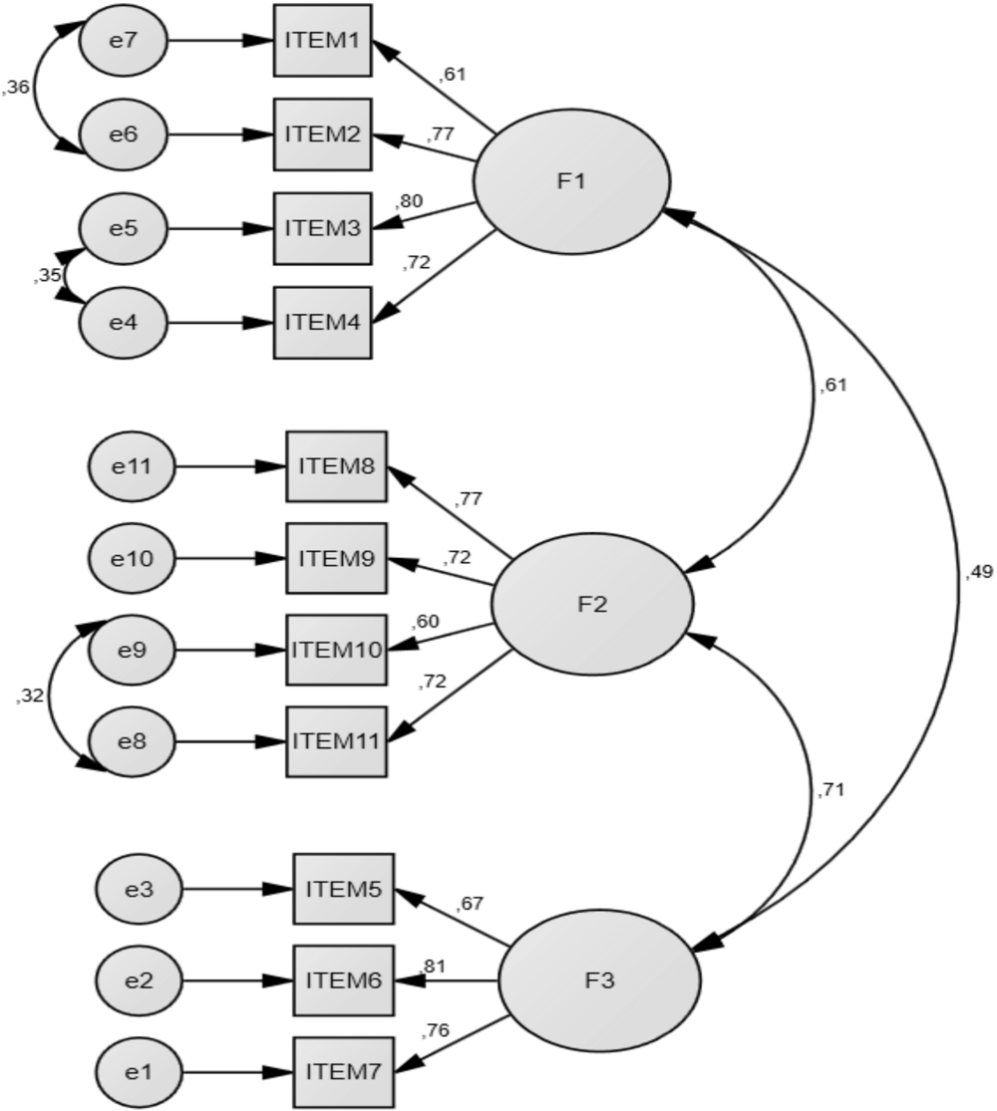
Confirmatory factor analysis of the Turkish version of the CSESSA scale.

The content validity was examined and revealed that all items had an I-CVI higher than 0.80 and the S-CVI Ave was 0.90. No items were removed from the scale. The CFA was performed using the three-factor structure of the original scale. CFA fit indices were found as χ2/df = 4.09, GFI = 0.95, CFI = 0.95, AGFI = 0.913, NFI = 0.940, TLI = 0.933, and RMSEA = 0.078 (Table 2). The results supported the construct validity of the three-factor model (Figure 2).

**Table 2.** Goodness-of-fit indices values of the scale (n = 510)

Fit Criteria	Good fit	Acceptable fit	Values
χ2/df	χ2/df <3	χ2/df <5	4.097
GFI	0.95≤ GFI≤1	0.90≤GFI<0.95	0.950
CFI	0.95≤ CFI ≤1	0.90≤CFI<0.95	0.954
NFI	0.95≤ NFI ≤1	0.90≤NFI<0.95	0.940
TLI	0.95≤ TLI ≤1	0.90≤TLI<0.95	0.933
AGFI	0.90≤AGFI≤1	0.85≤AGFI<0.90	0.913
RMSEA	0<RMSEA≤0.05	0.05<RMSEA≤0.08	0.078

Abbreviations: GFI, Goodness-of-fit index; CFI, Comparative fit index; NFI, Normed fit index; TLI, Tucker-Lewis Index; AGFI, Adjusted goodness-of-fit index; RMSEA, Root mean square error of approximation.

Cronbach’s alpha coefficients of the subscales were as follows: ‘husband/partner communication’ subscale (0.842), ‘provider communication’ subscale (0.770), and ‘choosing and managing a method’ subscale (0.810). Cronbach’s alpha of the total scale was found to be 0.867. The internal consistency of the total scale and subscales were adequate (Table 3).

**Table 3.** Item total correlation and Cronbach’s α coefficients of the scale

Items	Corrected item–total correlation	Cronbach’s α if item deleted
Item1	0.496	0.861
Item 2	0.591	0.854
Item3	0.599	0.854
Item 4	0.599	0.854
Item5	0.495	0.863
Item 6	0.578	0.855
Item 7	0.568	0.856
Item 8	0.646	0.851
Item 9	0.582	0.855
Item 10	0.521	0.860
Item 11	0.629	0.853
Total Scale Cronbach’s α = 0.867 Subdimensions Husband /partner communication Cronbach’s α = 0.842 Choosing and managing a method Cronbach’s α =0.810 Provider communication Cronbach’s α = 0.770		

The item-total score correlations of the scale ranged from 0.495 to 0.646. There was no significant increase in Cronbach’s alpha values when an item was deleted (Table 3). The test-retest correlation of the scale was found to be 0.83. The relationship between the total scale score and the subscales was investigated. A significant, positive and strong relationship was found between the total score of the scale and all its subscales (‘husband/partner communication’ p < 0.001, r = 0.729; ‘provider communication’ p < 0.001, r = 0.759; ‘choosing and managing a method’ p < 0.001, r = 0.797).

Hotelling’s T^2^ test was used to evaluate the response bias, and the Tukey Nonadditivity test was used to evaluate whether the scale had additivity. Hotelling’s T^2^ score was 92.349 (p = 0.001). Tukey’s non-additivity test was found to be p > 0.05, and the scale additive.

## Discussion

This study evaluated the psychometric properties of the Turkish version of CSESSA scale. Currently, no measurement tools are available for women’s contraceptive self-efficacy. The results confirm the validity and reliability of the scale. Furthermore, the results suggest that the CSESSA can be translated into other languages. This is significant, considering the limited number of validated international measures that comprehensively assess contraceptive self-efficacy.

The validity of a measurement tool means that it adequately measures its aims. In this study, independent translation and back-translation were performed, expert opinions were obtained, and a pilot test was conducted on a sample similar to the target population (International Test Commission, 2017). The results showed that the scale performed similarly to the original scale and provided cross-cultural validity (Souza *et al*., 2017). The content validity of the scale was examined using the Davis technique, and I-CVI and S-CVI/Ave were calculated. It is recommended that the minimum I-CVI value should be above 0.78 and the S-CVI/Ave value should be above 0.90 in the number of 6-10 experts (Polit and Beck, 2017). In this study, the I-CVI values for the items ranged from 0.80 and 1.00. The S-CVI/Ave value was determined as 0.90. Consequently, the Turkish version of the scale met the criteria for content validity (Almanasreh *et al.*, 2019; Esin, 2014). In this study, the CFA was performed to test the construct validity of the scale. CFA evaluates how many factors the scale has, which items are related to which factors, and whether the model fit index values are appropriate. These results indicate that the model had an acceptable fit (Boateng *et al.*, 2018; Byrne, 2016; Yaşlıoğlu, 2017).

Cronbach’s alpha coefficient, item-total score analysis, and test-retest analysis were used to evaluate the reliability of the scale. Cronbach’s alpha coefficient, which is used to evaluate the internal consistency of a scale, was found to be >0.70 in this study, which is considered acceptable reliability (Polit and Beck, 2017). The scale was found to be highly reliable with good internal consistency (Cronbach’s alpha = 0.867). All subscales were found to be either reliable or highly reliable. The item-total score correlation provides the discrimination index of each item (DeVellis, 2012). In this study, item-total correlation values were higher than 0.30 and varied between 0.495 and 0.646. These results show that the items accurately discriminated between participants. (Çokluk *et al.*, 2018; Streiner *et al.*, 2015). The test-retest correlation method was used to demonstrate the time invariance of the scale. This method is important because it helps to establish the reliability of the scale over time. The test-retest correlation of the scale is expected to be 0.80 (Nunnally & Bernstein, 1994; Weir, 2005). In this study, the test-retest correlation was calculated and found to be 0.83, indicating an acceptable limit. Hotelling’s T^2^ test was used to assess whether the scale items were perceived as identical by the participants and whether the responses were biased. When determining self-efficacy levels, the participants may have had biased responses. Therefore, evaluation of response bias is important for scale reliability (Seçer, 2018; Özdamar, 2016). The test outcomes indicated that the mean item scores differed and there was no response bias (Hotelling’s T2= 92.349, p = 0.001). These findings suggest that women do not exhibit bias when completing the scale. This scale comprises of three subscales. Tukey’s additivity test was used to assess the additivity of scale items. The test results showed that the scale items could be summed to obtain a total score (p > 0.05) (Özdamar, 2016).

The overall mean score of this scale was 9.03 (SD 1.51). In the original scale, the overall mean score was 8.72 (SD 1.72). Scores on the scale were high and skewed for both sample populations. This may be due to the high prevalence of modern contraceptives in the samples (73.3% for the Kenya sample and 67.3% for this sample) (Whiting-Collins *et al.*, 2020). The CSESSA scale assesses women’s self-efficacy in ‘husband/partner communication’, ‘provider communication’, and ‘choosing and managing a method’. This allows for a more comprehensive assessment of contraceptive self-efficacy. This scale is an appropriate tool for use by health professionals to evaluate contraceptive self-efficacy in women of reproductive age.

## Study limitations

This study also has some limitations. The data of the study were collected in a single region, and convenience sampling method was used. This may have affected the generalizability of the study.

## Conclusion

The Turkish version of the scale, a tool measuring contraceptive self-efficacy, is valid and reliable. This scale can assist health professionals in planning tailored interventions based on an individual’s specific needs and self-efficacy levels, with the aim of preventing unintended pregnancies.
